# Ecological and Temporal Constraints in the Evolution of Bacterial Genomes

**DOI:** 10.3390/genes2040804

**Published:** 2011-10-31

**Authors:** Luis Boto, Jose Luis Martínez

**Affiliations:** 1 Dpto Biodiversidad y Biología Evolutiva. Museo Nacional Ciencias Naturales. CSIC. C/JoseGutierrez Abascal 2. Madrid 28006, Spain; 2 Dpto. Biotecnología Microbiana. Centro Nacional de Biotecnología. (CNB-CSIC). Darwin 3. Madrid 28049, Spain; E-Mail: jlmtnez@cnb.csic.es

**Keywords:** bacteria, evolution, genomes, ecological constraints, horizontal gene transfer, endosymbiosis

## Abstract

Studies on the experimental evolution of microorganisms, on their *in vivo* evolution (mainly in the case of bacteria producing chronic infections), as well as the availability of multiple full genomic sequences, are placing bacteria in the playground of evolutionary studies. In the present article we review the differential contribution to the evolution of bacterial genomes that processes such as gene modification, gene acquisition and gene loss may have when bacteria colonize different habitats that present characteristic ecological features. In particular, we review how the different processes contribute to evolution in microbial communities, in free-living bacteria or in bacteria living in isolation. In addition, we discuss the temporal constraints in the evolution of bacterial genomes, considering bacterial evolution from the perspective of processes of short-sighted evolution and punctual acquisition of evolutionary novelties followed by long stasis periods.

## 1. Introduction

The classical theory of evolution is mainly based on the study of multicellular organisms. However, most of the biosphere is composed of unicellular microorganisms, which were not well known at the time when the theory of evolution came about. On the other hand, whereas experimental evolution studies are not easily achievable for multicellular organisms [1], due to the extensive time lapse required, these studies can be performed on microorganisms, which can present very large population sizes and extremely short generation times. Therefore, studies on bacterial evolution are of increasing interest to understand the basic mechanisms of evolution.

Bacterial experimental evolution studies as those performed by the groups of Lenski [2-7], Rainey [8-13], Levin [14,15] or Kolter [16-19] among several others, have served to experimentally test several general evolutionary processes, which would not be easily tested using multicellular organisms as models. These processes include sympatric diversification, punctuated evolution, kin selection, prey-predator interactions, bet-hedging or the effect of cheating on group selection among others.

Along with the studies on experimental evolution, the analysis on the *in vivo* evolution of microorganisms provides relevant information for understanding general aspects of the theory of evolution. Particularly relevant in this respect are the studies on the evolution of bacterial pathogens that produce long-lasting chronic infections. An example of this situation is the evolution experienced by *Pseudomonas aeruginosa* when this organism produces chronic infections. *P. aeruginosa* is an opportunistic pathogen that can colonize the lung of cystic fibrosis patients during decades and evolves during this colonization [20]. Studies on population biology dealing with the acquisition of antibiotic resistance by bacterial pathogens have also provided valuable information for understanding evolution [21].

The increased availability of the full genome sequences of prototypic strains of several bacterial species allows the detailed analysis of the differential effect that processes such as mutation and horizontal gene transfer (HGT) may have on the evolution of bacterial genomes. More recently, efforts have focused on sequencing several isolates belonging to the same bacterial species, in order to get a closer view to the process of bacterial diversification. These analyses, together with ecological studies that link the habitat of each species/isolate to its corresponding genomic ecotype might allow a more in depth understanding of the mechanisms driving bacterial evolution [22].

One important issue to be mentioned here is the fact that, in addition to the universal principle of evolution based in the selection of gradual modified descendants (mutants) claimed by Darwin [23] and the proponents of the Modern Synthesis [24], HGT, which allows fast, stepwise adaptation by quantum leaps [25], is a major evolutionary force in bacteria [26], and an example of punctuated evolution [27]. Of course, the acquisition of genes from other organisms can occur in all living beings, and indeed transposons were discovered in corn [28], but the relevance that HGT, as driver for acquisition of important adaptive traits [29-34], has on microbial evolution seems to be much higher than for other organisms [35-37].

Bacterial genome evolution is thus modulated by two main mechanisms: mutation (and recombination), which is common to the evolution of all living beings, and genome remodeling that results from gene acquisition and gene loss, and is more relevant for bacteria. It is important to note here that gene acquisition is only possible when microorganisms form part of communities, which contain members that may act as donors and recipients of the transferred elements. Mutation however is the unique mechanisms of variation for those organisms growing in isolation. Finally, gene loss is frequent for bacteria as endosymbionts that colonize a single ecosystem, where the physicochemical conditions are very constant through time.

In this article, we will review how these different processes contribute to the evolution of bacterial genomes (considering as bacterial genome both the chromosomal element and the mobilome or ensemble of mobile elements [32]), in relation to the different ecological conditions under which bacterial evolution occurs.

## 2. Tracking Phylogenetic Relationships in Bacteria

Molecular methods for tracking the phylogenetic relationship, and hence the evolution tree of organisms, are mainly based on the analysis of sequences of ortholog genes, being those encoding ribosomal RNAs the most popular to distinguish between species, to the point that this method has come to be considered the blueprint for reconstructing phylogenies [38]. However, whereas for higher organisms the trees generated using different orthologs are generally congruent, this is not necessarily so in bacterial species, where gene trees for different orthologs frequently show incongruencies [38,39] among them and with the aforementioned rDNA tree. HGT has been postulated to explain these incongruent trees and today; the acquisition of genes, plasmids and other genetic elements by horizontal gene transfer is accepted as an important mechanism for driving the evolution of bacterial genomes [35-37]. Evolution might be driven as well by gene duplication, divergence of paralogs and consequently genome expansion. Indeed, it has been suggested that this is an important mechanism for the evolution of myxobacteria [40], a group of social eubacterial predators characterized by the large size of their genomes [41]. Nevertheless, recent studies have shown that that this type of evolution is not very relevant in other bacterial species. As a consequence, it has been suggested that gene duplication and ulterior diversification of paralog genes play a much less important role in bacterial gene diversification than the acquisition of xenologs by HGT [42]. Altogether, this means that, although some vertical phylogenetic signal can still be obtained for microorganisms [43], genetic exchange has led to networks models when tracking evolutionary patterns [44].

The methods currently in use for distinguishing clones or populations in a given bacterial species can give insights into the relevance of HGT and mutation on these organisms. The golden standards for determining clonal relationships are pulse field gel electrophoresis (PFGE) and multilocus sequencing typing (MLST), each of which measures a different feature of bacterial genomes [45]. PFGE analyses the overall structure of bacterial genomes, and can therefore measure intragenomic recombination and gene trafficking (gene acquisition by HGT and gene loss), whereas MLST measures mutation in genes that are common to all members of a given species, otherwise known as the core genome [46]. One important aspect of these technologies with regards to bacterial evolution is the consistency of the attained results.

Bacteria have the outstanding capacity of modifying their genome either by mutation or by HGT. This capacity, together with the short generation times and considerably large populations of bacteria may enable considerable diversification. Indeed, bacteria colonize all known ecosystems in the biosphere, including extreme habitats. Consequently, bacterial species exhibit a great variety in the length of their genomes and in the type of genes they harbor. However, and despite this large ecological and genomic variability, the sequences of genes belonging to the core genome are usually greatly conserved for each bacterial species, even at the third position of the codons, which indicates that purifying selection is likely playing a relevant role in the evolution of bacterial populations. As will be discussed further on, short-sighted evolution [45,47] might be more able to justify the long lasting stability of bacterial core genomes than mutation clearance that is frequent in populations with sexual reproduction.

## 3. Sympatric Diversification Driven by Mutation and HGT

The presence of HGT-acquired elements in bacterial chromosomes makes the definition of bacterial species to be a fuzzy concept [22]. Because of this, different species concepts have been proposed for the bacterial world, among which is the proposal that ecotypes, defined as those bacteria presenting the same ecological behavior and a similar core genome, constitute valid taxonomic groups [48,49].

Whereas the core genome, that presents few changes among closely related bacteria, might define what is common, and thus the taxonomic root, ecotypes will define specific adaptations to particular ecosystems, a mode of sympatric diversification. Sympatric evolution of bacteria has been studied using experimental evolution models [50]. Furthermore, studies on the population dynamics of bacterial species based on the analysis of full-genome sequences allow to establish the role of sympatric diversification in the evolution of natural bacteria populations [51].

*In vitro* experiments have shown that the free-living bacterium *Pseudomonas fluorescens* can evolve rapidly when confronted with new environmental conditions to generate a repertoire of mutants that are capable of colonizing different habitats in a structured environment [9,11]. Since, during the experiment, bacteria grew in isolation, without any other counterpart that might be a donor of DNA, mutation is the only mechanism for achieving this diversification. It is important to note that the same types of mutants are selected when the experiment is repeated, which demonstrates that the adaptation process is not completely stochastic in the sense that adapting to the same ecosystem will involve the selection of the same variants.

These conclusions, derived from studies based on experimental evolution models, have been confirmed by the analysis of the *in vivo* evolution of *P. aeruginosa*, when this bacterial species colonizes the lungs of cystic fibrosis patients and presents a fast adaptation process [52-54]. People suffering from cystic fibrosis are frequently infected by *P. aeruginosa* and the same clone remains in the lung for decades. Since *P. aeruginosa* is an environmental microorganism, chronic infection requires the adaptation to a new ecosystem, in this case, the human lung. As in the case of the above mentioned *in vitro* experiment, *P. aeruginosa* diversifies into distinct populations as a consequence of mutations. Notably, the same set of mutations is found in isolates from different patients [55,56], and a similar adaptation is observed in patients suffering chronic obstructive pulmonary disease [57], indicating again that the process is not completely stochastic and that similar mutations may be expected to occur each time bacteria are confronted with same new environment. Overall this indicates that the processes of mutation and selection on their own may lead to the rapid diversification of bacterial populations.

Does this have a relevant implication in the long-term evolution of bacterial genomes? There is no simple answer to this question. In the case of free-living bacteria as those mentioned above, adaptation to a given environment would mean de-adaptation from another. In this situation, the stability of the genomes is guaranteed by periodic selection. On the other hand, diversification occasionally involves the emergence of cheaters, that are more fit than the evolved variant, but that can lead to the disruption of the whole community [9]. Because of this, in several occasions, fast mutation driven-diversification does not lead to long-term evolution, but is an example of short-sighted evolution (see below).

A different situation might take place when genes are transferred by HGT. In this case, bacteria can acquire a full set of proteins in a single step that enable them to colonize a new ecosystem. Nevertheless, and along with this potential adaptive advantage, the acquisition of new DNA confers a fitness cost to the new host because of the need to maintain, replicate, transcribe and translate the novel genetic elements [58,59]. Furthermore, the introduction of new proteins will require their adaptation to the host's metabolic and regulatory networks.

Under these circumstances, HGT-acquired genes will be rapidly lost unless they render relevant fitness benefits. Two examples that provide information on the trade-offs between fitness gain and fitness costs derived from the acquisition of novel genes by HGT are the acquisition of DNA conferring resistance to a toxic compound and the acquisition of DNA conferring the capability to colonize new environments. The first example has been explored with regard to antibiotic resistance. In an antibiotic-rich environment, bacteria are required to be drug resistant [60]. This means that when susceptible and resistant microorganisms are exposed to this type of strong selection, only the resistant ones will survive and the susceptible (parental) microorganisms will disappear. Nevertheless, since acquisition of DNA implies a fitness cost, it can therefore be predicted that once selection is over, the element conferring resistance will be lost, in a new example of periodic selection [21,61]. However, several plasmids have easy-to-get, hard-to-lose elements, either because they can contain toxin-antitoxin elements or because they harbor relevant elements that can be co-selected [21]. On the other hand, it has been demonstrated that bacteria can acquire mutations that compensate for the costs associated with resistance [62], which means that in habitats without the toxic selector agent, both susceptible and resistant bacteria can co-exist [63-65].

**Figure 1:**
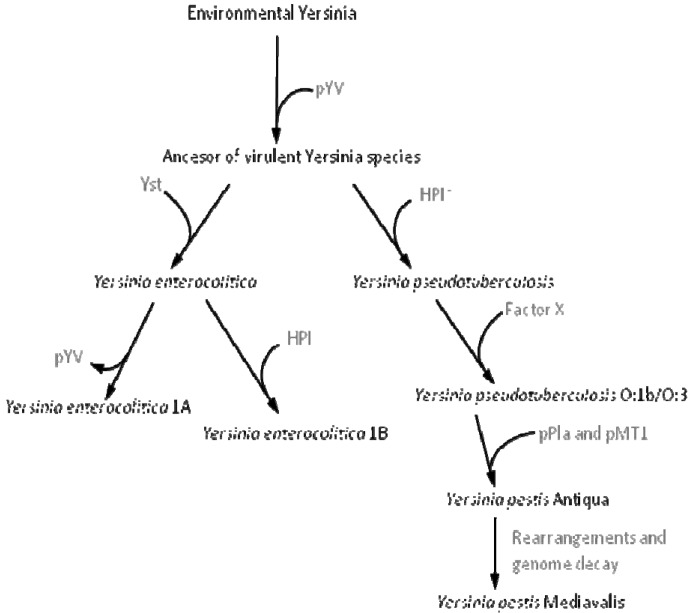
Evolution of *Yersinia*. The evolution of *Yersinia* exemplifies how the acquisition of exogenous DNA leads to evolution in *quantum leaps*. The acquisition of the plasmid pYV allowed the colonization of the human host, thus changing a non-virulent environmental Yersinia into the ancestor of the virulent members of this species. The incorporation of the pathogeneicity islands leads to the separation in two different evolutionary branches (*Y. enterocolitica* and *Y. pseudotuberculosis*) Yst and HPI*. The further incorporation of other elements as pPla and pMT1 enabled the evolution towards *Y. pestis*. The loss of elements such as that of the pYV plasmid by *Y. enterocolitica* 1A is also relevant for diversification. Finally, the stability of these elements into the *Yersinia* genome is enabled by the alteration of the former regulatory and metabolic networks to accomplish the best fit of the novel elements [69] into bacterial physiology. More details of this evolution process are shown in [72].

The situation observed when bacteria acquire DNA, which enables them to colonize a new ecosystem, might have a higher relevance for their long-term evolution. Indeed it has been shown that the acquisition and/or loss of DNA regions (genomic islands) not forming part of the core genome contributes to the diversification and adaptation of bacteria to colonize novel niches [29,31,33,66]. Since orfan genes and those coding hypothetical proteins are frequently specific for each bacterial species, it has been suggested that they might play a determinant role in the adaptation of the microorganisms to different habitats [67]. A good example of how the incorporation of novel DNA by HGT into a bacterial genome can trigger speciation is the acquisition of pathogenicity islands [29,34,68] by, *Yersinia pestis* [69-71], the cause of the plague. The genus *Yersinia* encompasses 15 species, three of which (Y. *pestis, Y. pseudotuberculosis* and *Y. enterocolitica*) are pathogenic to humans. All three pathogenic members of the *Yersinia* genus target the lymph tissues during infection because they carry the pYV virulence plasmid, which is needed for infecting these tissues and for overcoming the host defense mechanisms. This first event of HGT is the one that allowed the pathogenic *Yersinia* species to access a new habitat that lacked frequent bacterial competitors and was the initial *quantum leap* required for the speciation of these pathogens, making them able to access to a new niche (infected host). This evolution in *quantum leaps* has been followed by the incorporation of further elements, the loss of dispensable genes and mutations leading to the fine-tuning of the acquired determinants with the pre-existing bacterial regulatory and metabolic networks, all of which have led to the speciation of the *Yersinia* genus (see Figure 1 for details of this evolutionary process).

## 4. Evolution in Microbial Communities

Microbial communities, such as mammal gut microbioma or plants' rhizosphere, constitute dense and biodiverse ecosystems where different bacteria can support similar selective pressures. It is therefore not strange that in these communities, acquired genes conferring a relevant adaptive advantage can spread across the community members. This might be particularly important when the community must respond to the presence of a toxic compound such as an antibiotic. In these circumstances, each member of the community is required to be resistant, and the spread of antibiotic resistance genes will be favored by second-order selection processes [73,74]. This means that the introduction in a microbial community, in which members share the capability of exchanging DNA (genetic exchange communities [75]), of a given determinant that is useful for each of the members of the community, will lead to the presence of a strong selective force to the dissemination of the determinant among all members of the community. This might be the reason why genomes from coexisting microbes tend to be more similar than would be expected by chance [76]. For instance, some studies have recently shown that a high degree of interphyllum genetic exchange exists in Thermotogales or Aquificales bacterial groups that allow the widespread adaptation to shared environments between genealogically distinct bacteria [77,78]

Nevertheless, the acquisition of a new determinant by HGT does not necessarily imply its dissemination among all members of the population. This can be the case for the acquisition of genes encoding agarases or other carbohydrates present in algae and absent in terrestrial organisms by the human gut bacterium *Bacteroides plebius* in Japanese people [79]. This acquisition seems to have been mediated by the ingestion of marine bacteria associated to algal diet in the human population. Despite their acquisition by *B. plebius*, these elements have not disseminated among other microorganisms living in the same habitat. This may happen because, once a member of the community acquires the biodegradative characteristics required to use a novel resource, the stable food trade-offs already established among the population allow the use of this novel resource without the need of incorporating DNA by all members of the community. This possibility fits well with the idea that group selection, besides kin selection, might be an important evolutionary event in establishing cooperation among different organisms inhabiting complex, yet stable, communities [80].

The dissemination of HGT-acquired genes can be restricted by their epigenetic compatibility with the recipient genome. The products encoded by these novel genes need to be incorporated into regulatory and metabolic networks in recipient bacteria [73,81,82]. This process in not always easy (presence of similar products in the recipient network competing with the new product, problems in the expression of new genes, connectivity degree with other elements in the network, etc.), and this situation might impede the fixation of HGT-acquired determinants [81], in such a way that most new incoming sequences are rapidly eliminated [83]. However in some cases, acquired genes are of instant use [84], allowing recipient bacteria to instantaneously exploit the evolutionary novelty provided by the new elements acquired by HGT. The trade-offs between fitness costs (including epigenetic compatibility) and fitness gain, understood as the capacity to exploit a new habitat, constitute a major bottleneck for the fixation of HGT-acquired genes.

## 5. Free-Living Bacteria, When Size Matters

Free-living bacteria are those capable of colonizing a variety of ecosystems. For these bacterial species, two different types of evolution can be foreseen. In the first, all members of each given species are able to colonize the different habitats, which means that this bacterial species will have large core genomes and limited accessory ones.

This is likely to be the situation for *P. aeruginosa*, a bacterial species capable of colonizing a variety of terrestrial and aquatic habitats, and producing infections in different hosts ranging from plants to humans. It is noteworthy that the same virulence determinants required to infect plants serve to infect humans [85]. This suggests that all members of these bacterial species have the capacity of colonize environmental and clinical ecosystems [86]. The detailed genetic analysis of several isolates from different ecosystems has shown that indeed there is not a clear cut-off between environmental and clinical isolates of this bacterial species [45,87].

Furthermore, the genome of *P. aeruginosa* is large and enriched in sensory and regulatory elements [88], indicating the need of sensing different ecosystems and respond accordingly by all members of the species. This population structure does not mean however that *P. aeruginosa* is genetically static. Although the core genome is large and presents an overall conserved synteny, a few loci are still subject to diversifying selection. These loci present a non-random association of genotypes, such as defining clonal regions within the genome. It is important to notice that different genotypes are associated to specific repertoires of accessory genes, showing that the association of specific clones to given accessory elements is an ancient event in the evolution of *P. aeruginosa* [45,87], and does not seem to be particularly relevant towards enhancing the capacity of specific clones to colonize a given ecosystem.

In sharp contrast to this situation, genome analysis of several *E. coli* isolates shows a different type of evolution. In this bacterial species, the core genome is much smaller than that of *P. aeruginosa* and the accessory genome is much larger [89]. Furthermore, whereas some *E. coli* strains are commensals, some others are pathogens, and the differences between one and the other mainly rely on the elements acquired by HGT. This genetic structure of *E. coli* populations allows defining this bacterial species as a multi-specialist in the sense that the whole species can colonize different ecosystems, but each lineage has acquired a specific genetic repertoire, allowing its specialization for colonizing a given environment.

A more drastic example of this situation concerns the plankton components *Prochlorococcus marinus* and *Pelagibacter ubique* [90-92], which are supposed to be among the most abundant components of the biosphere. These organisms present extremely compact genomes, a characteristic that is common for intracellular endosymbionts but supposed to be rare in free-living organisms, because the genomic reduction observed in free living bacteria cannot easily be explained by genetic drift [93], as is the case for intracellular endosymbionts (see below). The study of different isolates of *Prochlorococcus*, suggests that they diversify to occupy a variety of oceanic environments with different light, temperature and salinity conditions. However, despite the authors statement that the observed genomic shrinkage and the existence of different lineages of *Prochlorococcus* are not remnants of genetic drift, but potential outcomes of a niche-oriented stepwise diversification, the reasons for gene-loss are still unclear. One possibility might be that the physicochemical conditions of each of these niches may be as stable as those of endosymbionts and under these conditions, a bacterial autotroph, not requiring as much metabolic versatility as an heterotroph, would have evolved towards genome reduction. In a nutrient-poor (mainly in N and P elements) environment such as sea surface water, the replication, transcription and translation of unnecessary genes is costly and therefore natural selection will favor the deletion of these genes.

The constant increase in the number of genome sequences enables the estimation of the size of core genome, which is defined as the gene repertoire that is common to all members of a given bacterial species and the so called pan-genome [37,94], defined as the full genetic repertoire of this species. It is important to mention that the core genome and the accessory genome frequently present different G+C composition, which indicates a different phylogenetic origin [46].

**Figure 2:**
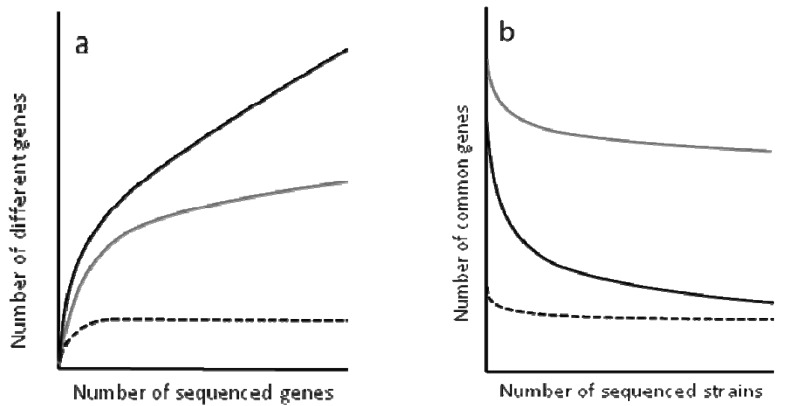
Open and closed bacterial genomes. By sequencing different isolates from the same bacterial species, it is possible to distinguish between open and closed genomes. The Figure shows models of open and closed genomes based on data from [99,100]. For instance, panel a shows that after sequencing four *Buchnera aphidicola* isolates, sequencing a new more only provide repeated (already sequenced) genes (dotted line), indicating that this species harbors a closed genome. However, the sequence of more isolates from *P. aeruginosa* or from *E. coli* allows the increase in the number of genes. This increase is higher for *E. coli* (black line) than for *P. aeruginosa* indicating that the genome of *E. coli* is more open than for *P. aeruginosa*. As shown in panel b, presenting a very open genome might mean that the core genome is small. Black: the core genome of *E. coli*; Grey: the core genome of *P. aeruginosa*; Dotted line: the core genome of *B. aphidicola*. The Figure was drawn to represent the concept of open and closed genomes and is based on the data presented in [99].

To determine the complexity of the pan-genomes, the results are analyzed using rarefaction curves (Figure 2). These curves allow the distinction between closed and open genomes [94-98]. The former are those presenting a non-asymptotic curve, which means that upon sequencing a given number of genomes, the number of novel genes does not increase. One example of closed genome is the endosymbiont *Buchnera aphidicola* (see the next section for a discussion of evolution in endosymbiotic bacteria). This bacterial species occupies an isolated and restricted niche that hampers its possibilities of acquiring new genes and the sequence of four strains has shown that any novel isolate to be sequenced would not contain any different gene to those that have already been found in analyed genomes.

In contrast to the situation observed for *B. aphidicola*, the pan-genomes of several bacterial species present asymptotic rarefaction curves, indicating that the number of genes that can be shared by the population is theoretically infinite. However, the shape of the curve is not the same for all genomes and can provide an estimation of the size of the pan-genome. For instance, each new sequenced strain of *Streptococcus pneumoniae* adds around 30 genes to its pan-genome, and the number decreases with each new sequenced strain, whereas each new strain of *E. coli* adds over 300 genes to the its pangenome. Based on these analyses, it has been predicted that the pan-genome of *S. pneumonia* encompasses around 3300 to 5000 genes, 1647 of which make up its core genome [100]. This likely reflects that *S. pneumoniae* colonize a restricted habitat, but is not in isolation as *Buchnera*.

## 6. Bacteria Living in Isolation

Not all bacteria live in communities. Bacteria inhabiting either deep or poor soils, pathogens causing infections in otherwise sterile habitats (such as blood) or insect endosymbiotic bacteria live in relative isolation from other bacteria. In these circumstances, the possibilities of gene exchange are low and bacterial genome evolution is determined mainly by mutation or by genome rearrangements [101], including genome reductive evolution.

One important issue concerning mutation-driven evolution is to know whether adaptability itself can evolve [102]. In this regard, it has been shown that toxic agents such as antibiotics can transiently increase mutation rates and hence help to select resistant strains [103-105]. Increasing mutability under stress is still a controversial issue. Some models indicate that bacteria under stress (stationary phase, antibiotics) can undergo gene amplification [106-109], which is resumed upon stress removal. Other models however postulate that increased mutability under stress is a tightly regulated process [110] that involves the production of error-prone DNA polymerases and the induction of the SOS system among other processes [111,112]. Independently of which model best explains these results, it seems that mutation itself is not a fixed value and can be regulated depending on bacterial growing conditions.

The description of bacterial strains (mutators) presenting much higher mutation rates than those of the overall population [113,114] indicates that mutability is a selectable trait that can drive the evolvability [102,115] of bacterial populations. Indeed, the percentage of hypermutator strains is higher in the case of bacteria causing chronic infections, as described in [57,116,117]. This enrichment is supposed to be as a consequence of a second-order selection process by which those isolates with higher probability of acquiring antibiotic resistance (mutators) in the treated patient are selected [118]. The finding that co-evolution with viruses [119] increases in bacterial population the fraction of isolates presenting high mutation rates, further supports the idea that elevated stress levels select for mutators through second-order selection processes.

Besides mutations, in a scenario lacking DNA donors, genome rearrangements emerge as a source of novelties, leading to operon shuffling and the emergence of new operons [120] or association of different mobile elements in order to generate novel integrative elements such as multi-resistance determinants [121].

Mutations are relevant in processes of transient adaptation as those experienced by human pathogens producing chronic infections and in ensuring stable adaptation leading to long lasting evolution processes and eventually speciation as those observed in the case of endosymbionts. In the first case, the adapted strain leaves the infected host and must compete with their non-evolved counterparts. Under these circumstances, it is expected that the adapted mutant strain will be outcompeted and mutations will not be fixed (short-sighted evolution [47]). This situation has been theoretically discussed and a source-sink model for explaining the dynamics of virulence in opportunistic pathogens have been proposed, in which the evolution of such pathogens can be considered from the standpoint of continuous switching between permanent environmental (source) and transient infective (sink) habitats [122]. It is important to mention here that this mode of evolution is only possible when the opportunistic pathogen already harbors the determinants required for producing the infection in the compromised patient. Otherwise, a first step allowing access to the new niche will be required for the formerly non-pathogenic bacteria (see discussion on *Y. pestis* above). An exception for this situation may occur when the mutations allow epidemicity or are acquired by an epidemic clone that is already well adapted to clinical settings, in which case the clone can be maintained in the clinical setting, without competing with their counterparts that live in the species'natural habitats, and mutations can be fixed. However, this situation, although described for some *P. aeruginosa* clones [123], is infrequent.

In the second evolutionary process, mutation enables access to the resources of a novel environment or is secondary to the acquisition by HGT of the elements required for entering in this ecosystem. This means that, once the organism have acquired the required capacity to colonize a given ecosystem, the optimization of its metabolism is triggered towards reducing fitness costs that results from the presence in its genome of un-needed genes, and therefore leads to genome reduction [124]. This situation is observed in intracellular bacteria, especially in endosymbionts. The access to the intracellular milieu is stressful and because of this, few organisms can colonize this habitat. However, once this habitat is colonized it becomes a rather stable environment in which no other competitors are present. Insect endosymbiotic bacteria are an example. They exhibit highly derived genomes characterized by an important genomic reduction accompanied by a biased base composition toward A/T rich genomes as a consequence of their particular life mode and host dependence [125,126]. An extreme case of genomic reduction is observed in the genome of *Carsonella rudii*, that harbors just 182 open reading frames and exhibits the total loss of genes for numerous categories [127].

Genome shrinkage is mainly observed in obligate endosymbionts, whose genomes exhibit a high genomic reduction, an important structural stasis and high sequence evolution rate [126,128], whereas facultative endosymbionts exhibit more dynamic genomes and more gene conservation. In addition, facultative symbionts have more repetitive sequences and mobile elements than obligate symbionts, which enable the former to have higher gene plasticity.

Genome reduction follows a temporal pattern in which gene loss is fast during the first stages of the evolutionary process, diminishing afterwards and leading to stasis with few changes in the genomes once optimal adaptation is reached. One important aspect is that, besides reduction, some other changes that allow a better adaptation can be observed. For instance, the amplification of genes encoding the pathways for tryptophan and leucine biosynthesis in *Buchnera* [129] constitute an example of adaptation to improve provisioning of host nutrients that involve genome expansion, and not genome reduction. Although the final structure of the genome would be very similar if the process is repeated because the same unnecessary genes would be eliminated, the evolutionary trajectories might present some degree of stochasticity, with genes being eliminated in a different order. In this regard, small population size determines a reduction in the role of natural selection in endosymbiotic bacteria and hence genetic drift modulates genome evolution in the endosymbiotic scenario driving to the fixation of mildly deleterious mutations (for example amino acid changes that reduce the stability of proteins), and the elimination of nonessential genes [130]. Nevertheless, recent theoretical and experimental studies indicate that gene loss is not completely random and that metabolic constraints play a relevant role in the evolution of genome reduction of bacteria endosymbionts, indicating that both random gene drift and natural selection are partners in the process of endosymbionts evolution [81].

## 7. Homologous Recombination and Microevolution in Bacteria

In previous sections we have reviewed the main forces shaping the evolution of bacterial genomes as a function of the different ecosystems that bacteria might face. In addition to the different forces discussed, homologous recombination also plays an important role in bacterial evolution, allowing the exchange between closely related bacteria of small genomic regions. Two types of homologous recombination can be important for the evolution of bacterial genomes. One is when recombination occurs in a given genome without acquiring DNA from another cell (intragenomic recombination). Here, the process might produce genome reorganization and gene duplication. The other type of homologous recombination happens when a recipient cell acquires, by HGT, DNA presenting a large degree of similarity with its own genome from closely related bacteria.

Intragenomic homologous recombination can be important in the evolution of paralog genes derived from gene duplication and indeed it has been demonstrated that genome expansion of mixobacteria is driven by this mechanism (see above). However, despite several studies [131,132] suggesting a role for gene duplication events in the evolution of bacterial genomes, few duplicated genes are found in phylogenetically controlled studies for most bacterial species, and recent studies indicate that gene acquisition by HGT, more than duplication, drives functional diversification of protein families in bacteria [42,133], and hence colonization of novel ecosystems.

Another possible effect of intragenomic homologous recombination could be the generation of deletions by recombining repetitive sequences. However, studies on genome size reduction by experimental evolution suggest that extensive bacterial genome reduction can occur on a short evolutionary time scale and that homologous recombination only plays a limited role in this process [134].

Although homologous recombination between different individuals harboring different alleles of the same gene is supposed to be a mechanism of exchange of gene variants more than a mechanism for the acquisition of evolutionary novelties, it can also introduce genetic variation without the need of gene duplication One example of this situation is the development of resistance to beta-lactam antibiotics by *Streptococus pneumoniae* and *Neisseria ghonorroeae* due to the acquisition of DNA from closely related bacteria and its recombination, which allows the formation of antibiotic resistant mosaic Penicillin Binding Proteins [135,136]. Homologous recombination between members of the same species can also be an important mechanism modulating the bacterial evolution [137]. For instance, it has been proposed that the evolution of virulent *E. coli* clones is driven by the acquisition of pathogenicity islands, which, as in the case of *Y. pestis*, permits access into a new niche (infected patient), followed by homologous recombination in some parts of the genome [29,137-139] likely to evade the host immune response by generating antigenic variability, a feature described in other bacterial pathogens like *Mycobacterium tuberculosis* [140].

Since homologous recombination only occurs between very closely related DNA sequences, it has been suggested that the barriers to homologous recombination can be suitable markers for the classification of bacterial species [141-143]. This implies that restrictions to homologous recombination can contribute to the speciation process in bacteria [22,48]. In this regard, the finding that different clonal complexes of *Salmonella enterica* present restrictions for recombining has been interpreted as evidence that these lineages might represent incipient species [144].

Besides contributing to diversification, homologous recombination contributes, as in individuals with sexual reproduction, to convergent evolution and to the buffering of deleterious mutations. One example of this statement can be found in the recombination between *Salmonella paratyphi* A and *Salmonella typhi*, which has allowed the exchange of gene variants that are important for their adaptation to a common ecological niche, which is the human host [145].

One final aspect by which homologous recombination influences the evolution of bacteria is through its role in the combination of the different modules that form a mobile genetic element, which is transferred as a whole. One good example of this situation is the evolution of integrons [146]. These elements consist of arrays of gene cassettes, each one flanked by imperfect inverted repeats (*att* regions). Recruitment of new gene cassettes is due to the site-specific recombination [147] of the *att* regions, a process mediated by an integrase, also present in the integron. In recent years, several integrons, presenting different arrays of antibiotic resistance genes that are co-transferred have been described, showing that homologous recombination of short sequences flanking non-homologous genes can be a source of adaptability [148]; in this case towards the selective pressure imposed by antibiotics belonging to different structural families [149].

One important issue to discuss here is the fact that homologous recombination can be relaxed under certain conditions, thus allowing the interchange of genetic material between bacterial strains belonging to different species. This is mainly important in the case of mutator strains presenting defects in the mismatch repair (MMR) system, which present both high mutation rates (see above) and less stringent homologous recombination [150-152]. This indicates that the mutator phenotype will favor fast adaption by both increasing mutation rates and recombination. The finding that MMR genes present higher sequence mosaicism as compared to housekeeping genes in different *E. coli* lineages correlates with the hyper-recombination phenotype of MMR-deficient mutators, and supports a mechanism of evolution that involves modulation of mutation and recombination rates by recurrent losses and reacquisitions of MMR gene functions [153].

Considering its role in bacterial speciation, homologous recombination is a force most likely acting in the different ecological scenarios discussed in this review

## 8. Temporal Constraints in the Evolution of Bacterial Genomes: Punctuated Equilibrium and Short-Sighted Evolution

All along the review, we have stated that bacteria present an impressive capacity of adaptation to any given change. This adaptation is given by their high population sizes and the strength of the processes that allow diversification, which include mutation, homologous recombination and HGT. The enhanced mutability and recombination shown by bacteria under stress, as well as the existence of strains presenting high mutation rates (mutators), increases the capacity of variation of bacterial genomes and consequently the adaptability of bacterial populations. This high adaptability potential implies that when bacteria face a new selective force, their evolution should be fast. Indeed, information on the evolution of bacterial pathogens after the discovery of antibiotics indicates that this is true. A few decades after introducing antibiotics to therapy, bacteria have acquired a variety of resistance mechanisms and resistant organisms, which were susceptible before the use of antibiotics, became widespread [64,65,154-156]. However, despite this fast adaptation, bacterial core genomes are rather stable (see above), and it can be stated that the evolution of bacterial genome is an example of punctuated evolution in which periods of fast evolution are followed by stasis [27]. The periods of fast evolution are associated with the colonization of a new habitat [157] or with the presence of a new selective force. Stasis occurs after this process of fast adaptation, unless the ecosystem/selective force changes again. Following on from the example of the antibiotics, a paradigmatic example of this situation is the history of the TEM 1 β-lactamase that confers resistance to first generation β-lactams. This plasmid-encoded enzyme was rapidly disseminated in plasmids among *Enterobacteriaceae* (fast evolution) and remained without any change in the population (stasis) until inhibitors of its activity were launched into the market (novel selective force), i.e., the moment at which a strong allelic diversification occurred [158].

**Figure 3:**
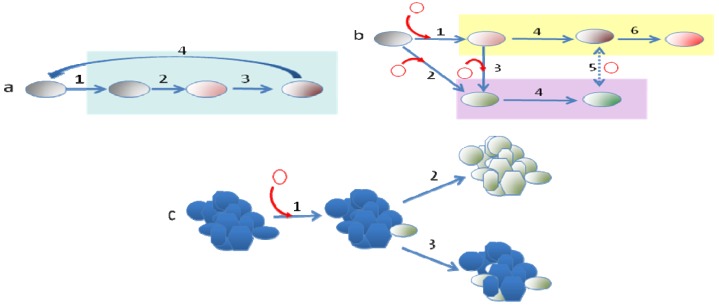
Summary of forces modulating bacterial evolution. The Figure shows the main forces driving the evolution of bacterial genomes: (**a**) Some organisms, such as the opportunistic pathogens of environmental origin, can colonize different habitats. Nevertheless, when an organism enters in a new ecosystem (1) where there are no DNA donors, as occurs in some infections (green box), mutation and gene rearrangements, including gene duplication, gene loss and genome translocation, which are triggered by homologous recombination (2,3) are the only sources of gene variation. These modifications can produce the de-adaptation from the initial habitat in such a way that if this evolved organism returns to its original environment (4), it will be outcompeted by the bulk of the population and these adaptive changes will not be fixed (short-sighted evolution). Nevertheless, if the new habitat is stable and the bacteria do not return to their former environment, the changes may be fixed. (**b**) The acquisition of DNA (1, 2) by HGT (red circles in the figure) might allow, in a single step, the acquisition of the abilities required to colonize a new habitat (yellow box), a process that has become known as evolution in *quantum leaps*. After entering in this new ecosystem, the bacteria can further evolve by acquiring novel DNA elements (3), which enable the colonization of yet another ecosystem (purple box). This first step is followed by the fine-tuning of the bacterial networks through mutation and recombination using the same processes described in (a). Homologous recombination can lead to convergent evolution if the divergence of the genomes is not excessively high (5). In the case of bacteria growing in isolation in a very stable ecosystem, genomes evolve towards their reduction (6). (**c**) The acquisition by a member of a stable community of DNA that confers a fitness advantage (1) can be followed either by its distribution (green bacteria) among all members of the community (2) if this DNA confers an independent advantage (antibiotic resistance in the presence of antibiotics) or by its maintenance just in some members, without transferring to others (3) if the advantage acquired by one member is sufficient to increase the fitness of all the community (as is the ability to use a novel food resource, see text).

Another aspect to discuss here is whether the different processes of evolution follow a temporal pattern. This seems to be the case in the evolution of bacterial pathogens that begin with the acquisition by HGT of the elements that allow entrance into the new host, and the consequent spread in this novel niche of this evolved clone (clonal expansion), followed by the acquisition of other elements that might work to produce different types of infections and the fine-tuning of the bacterial physiological networks, driven by mutation and homologous recombination, to allow its adjustment to the conditions of the new host and permit a good integration of the newly acquired determinants (Figures 1 and 3). When the trade-offs of cost-benefits for accessing the new host impede the return of the evolved bacteria to its original habitat, a strong genome reduction by purifying selection can be foreseen [93]. The most drastic reduction in genome size occurs in obligate mutualistic endosymbionts, which form a common metabolic network. Although, it has been described that the process of gene loss has no clearly defined limit [159], most changes occur in the first stages of adaptation to a new habitat.

A final aspect of the temporal constraints of bacterial evolution, regards adaptive changes that are not fixed and, thus, do not contribute to the long-term evolution of bacterial populations. This situation has received the name of short-sighted evolution and reflects the fact that bacteria can evolve to better adapt to a given niche, but, if this adaptation represents de-adaptation to the original ecosystem and the new habitat is not stable enough to allow long-term evolution, the changes are not fixed and thus constitute futile adaptation cycles in the evolution of otherwise rather stable bacterial genomes [122].

Unlike what happens in other organisms presenting smaller population sizes, even if the adaptation of a given bacterial strain to a specific habitat compromises its fitness in the previous ecosystem, this situation will not compromise the fitness of the overall population, which is by far much larger. Because of this, short-sighted evolution is a fruitful mechanism of bacterial adaptation that allows the adjustment of a given strain to a specific niche just during time-lapse in which the strain colonizing this habitat, despite the fact that the evolutionary novelty is not fixed [47].

## 9. Concluding Remarks

An emergent view of evolution is that a plurality of mechanisms and processes drive the diversification of living beings. The study of bacterial evolution has contributed to reinforce this idea, showing the coexistence of multiple mechanisms (gene modification, gene gain and loss, genome rearrangement, utilization of genetic available resources, homologous recombination, etc.) driving microevolutionary processes and long term diversification of bacteria (Figure 3). The relative importance of these mechanisms for bacterial evolution is determined by the ecological scenarios in which bacteria live and follows specific temporal patterns such as those driven by punctuated equilibrium and short-sighted evolution.
